# *MiR-27a* rs895819 is involved in increased atrophic gastritis risk, improved gastric cancer prognosis and negative interaction with *Helicobacter pylori*

**DOI:** 10.1038/srep41307

**Published:** 2017-02-02

**Authors:** Qian Xu, Tie-jun Chen, Cai-yun He, Li-ping Sun, Jing-wei Liu, Yuan Yuan

**Affiliations:** 1 Tumor Etiology and Screening Department of Cancer Institute and General Surgery, the First Affiliated Hospital of China Medical University, and Key Laboratory of Cancer Etiology and Prevention (China Medical University), Liaoning Provincial Education Department, Shenyang 110001, China; 2 Department of Molecular Diagnostics, Sun Yat-Sen University Cancer Center, State Key Laboratory of Oncology in South China, Collaborative Innovation Center for Cancer Medicine, Guangzhou, China

## Abstract

*MiR-27a* rs895819 is a loop-stem structure single nucleotide polymorphism affecting mature miR-27a function. In this study, we performed a comprehensive analysis about the association of rs895819 with gastric cancer risk and prognosis, atrophic gastritis risk, as well as the interactions with environmental factors. A total of 939 gastric cancer patients, 1,067 atrophic gastritis patients and 1,166 healthy controls were screened by direct sequencing and MALDI-TOF-MS. The association of rs895819 with clinical pathological parameters and prognostic survival in 357 gastric cancer patients was also been analyzed. The rs895819 variant genotype increased the risk for atrophic gastritis (1.58-fold) and gastric cancer (1.24-fold). While in stratified analysis, the risk effect was demonstrated more significantly in the female, age >60y, *Helicobacter pylori (H. pylori*) negative and non-drinker subgroups. Rs895819 and *H. pylori* showed an interaction effect for atrophic gastritis risk. In the survival analysis, the rs895819 AG heterozygosis was associated with better survival than the AA wild-type in the TNM stage I–II subgroup. *In vitro* study by overexpressing miR-27a, cells carrying polymorphic-type G allele expressed lower miR-27a than wild-type A allele. In conclusion, *miR-27a* rs895819 is implicated as a biomarker for gastric cancer and atrophic gastritis risk, and interacts with *H. pylori* in gastric carcinogenesis.

Single nucleotide polymorphisms (SNPs) are common variations of the hereditary factors thought to be associated with several forms of cancers^1^,^2^. MicroRNAs (miRNAs) SNPs are located in the pre-/pri- or mature miRNAs, which could change the binding site with transcriptional factors or Drosha/Dicer enzyme and thus affect the quality and quantity of mature miRNAs^3^,^4^. *MiR-27a* contains two SNPs, rs895819 and rs11671784, with rs895819 being located in the 6 bp downstream of rs11671784 on chromosome 19p13.13^2^. The *miR-27a* rs895819 polymorphism is an unusual miRNA-SNP due to its location in the coding region of the pre-mir-27a hairpin in the stem-loop, which could be cut by Dicer in the process of pre-miRNA maturation^5^. Recent studies speculated that the A → G change of rs895819 could shorten the stem-loop structure and affect the processing of miR-27a^6^,^7^, suggesting that rs895819 was a functional SNP. The results of previous investigations concerning the association between this polymorphism and cancer risk are controversial. For example, several studies reported the variant allele could decrease the cancer risk^7^,^8^,^9^, while another study found the AG and GG genotypes increased the risk of gastric cancer^10^, and the others suggested that the association between the variant G allele and cancer risk was statistically insignificant^11^,^12^,^13^. However, for the most important of all, it was reported that direct sequencing or MALDI-TOF Mass-ARRAY using primer extension from one direction (not overlapping with any SNP) is essential for investigations of rs895819 and rs11671784^14^.

Gastric carcinogenesis is influenced by multiple hereditary and environmental factors, and epidemiological studies have suggested that individual hereditary susceptibility affects the incidence of gastric cancer^15^. Recently, accumulating evidences have demonstrated environmental factors such as smoking, drinking and *Helicobacter pylori (H. pylori*) infection, are all critical risk factors for gastric carcinogenesis. In addition, environmental factors and gene polymorphisms may also be involved in gastric carcinogenesis. Helicobacter pylori (*H. pylori*), as one of the important factors for gastric adenocarcinoma and mucosa-associated lymphoid tissue lymphoma, are able to result in chronic infection^16^. A synergistic effect of *H. pylori* infection with gene polymorphisms could contribute to the development of gastric cancer^17^,^18^,^19^. However, it is still unclear whether an interaction effect exists between miR-27a rs895819 SNP and *H. pylori* for gastric carcinogenesis, of which the exploration will help to the comprehension of the carcinogenic biology.

In this study, sequencing and MALDI-TOF-MS were used as the accepted detection technology to perform a comprehensive analysis for this special SNP, including the association with risk and prognosis, cancerous and precancerous status, and interaction effects with the environmental factor *H. pylori*.

## Results

### Patient characteristics

Two-stage study was conducted: in stage 1, the samples came from 724 retrospectively recruited individuals, including 215 gastric cancer patients, 205 atrophic gastritis cases and 304 controls, which attended the First Affiliated Hospital of China Medical University between 2005 and 2010. In stage 2, a total of 2,448 cases were included, consisting of 724 gastric cancer cases, 862 atrophic gastritis cases and 862 matched healthy controls from the Zhuanghe Gastric Diseases Screening Program or from patients who attended the First Affiliated Hospital of China Medical University between 2002 and 2013.

The baseline characteristics of the subjects in the control, atrophic gastritis and gastric cancer groups were listed in Supplementary Table S1. No significant difference was found in terms of age and sex between the case and control groups. In stage 2, the subjects were selected from two different sources, then we analyzed the baseline of the subjects and the distribution frequency of rs895819 and no significant difference was discovered between the two groups (Supplementary Table S2). The characteristics of the subjects in the intestinal-type and diffuse-type gastric cancer subgroups were listed in Supplementary Table S3.

### Frequencies of miR-27a rs895819 and rs11671784 determined by direct sequencing

Direct sequencing of *miR-27a* rs895819 in the 724 samples revealed 392 cases of the AA genotype, 291 cases of the AG genotype and 41 cases of the GG genotype. Among the 724 samples in the control group, only one individual carried the rs11671784 AG genotype, while the other 723 samples all carried GG genotype.

### Association of miR-27a rs895819 with gastric disease risk

The *miR-27a* rs895819 SNP was found to meet with Hardy–Weinberg equilibrium (*P* > 0.05, Table 1). Rs895819 was associated with an increased risk for atrophic gastritis in stage 2 (GG vs. AA: *P* = 0.045, OR = 1.58; GG vs. AG + AA: *P* = 0.045, OR = 1.56). We also found rs895819 was associated with an increased risk of gastric cancer in the analysis of merge data (AG vs. AA: *P* = 0.027, OR = 1.24; AG + GG vs. AA: *P* = 0.043, OR = 1.21; Table 1). In addition, the method suggested by Thakkinstian *et al*.^20^ was employed to select the optimal genetic models for rs895819 SNP including dominant model, recessive model, codominant model and complete overdominant model. In the merged data, for the atrophic gastritis risk, OR1, OR2 and OR3 were 1.30 (*P* = 0.174), 1.02 (*P* = 0.797) and 1.25 (*P* = 0.248), respectively, which was in accord with the principle of OR1 > OR2 > 1 and OR1 > OR3 > 1, indicating a codominant model effect (Table 1). For the gastric cancer risk, OR1, OR2 and OR3 were 1.03 (*P* = 0.906), 1.24 (*P* = 0.027) and 0.81 (*P* = 0.318), respectively, which was in accord with the principle of OR2 = 1/OR3 ≠ 1 and OR1 = 1, indicating a complete overdominant model effect (Table 1). Thus, for the gastric cancer risk, in the complete overdominant model, AG heterozygote was associated with an increased risk of gastric cancer compared with GG + AA genotypes (*P* = 0.025, OR = 1.24, Table 1).

When the gastric cancer group was divided into intestinal-type and diffuse-type according to Lauren classification, no association was found between diffuse-type subgroup and controls, which was also fit for intestinal-type subgroup though the dominant model closely reaching statistical significance (GG vs. AA, *P* = 0.050, OR = 1.32; Supplementary Table 4).

In the stratified analysis, the heterogeneity test was performed for the host’s characteristics, and the results were shown in Supplementary Table S5. Both the age factor for atrophic gastritis vs. control group and the *H. pylori* infection factor for gastric cancer vs. control group almost reached statistical significance (*P*_heterogeneity_ = 0.095 and 0.078, respectively). Thus, the stratified analysis for all the host’s characteristics was conducted. In the female subgroup, rs895819 was found to be associated with an increased risk of atrophic gastritis and gastric cancer (atrophic gastritis risk: GG vs. AA, *P* = 0.030, OR = 1.86; gastric cancer risk: AG vs. AA, *P* = 0.032, OR = 1.44). In the age >60y subgroup, individuals carrying with the AG heterozygosis were associated with an increased gastric cancer risk (AG vs. AA, *P* = 0.001, OR = 1.74). In the *H. pylori-negative* subgroup, the GG genotype was associated with an increased risk when compared with the AA wild-type (*P* = 0.047, OR = 1.62). In the non-drinking subgroup, individuals with the GG variant genotype were associated with increased atrophic gastritis risk (GG vs. AA, *P* = 0.037, OR = 2.08, Table 2).

### Interaction of rs895819 and H. pylori in the risk of gastric cancer/atrophic gastritis

More significant association was found in the H. pylori subgroup with the risk of gastric cancer and atrophic gastritis compared with that of the total samples. Therefore, we analyzed the interaction effect between rs895819 and *H. pylori* infection status. The result showed a negative interaction for atrophic gastritis risk (*P*_interaction_ = 0.013, OR = 0.54; Table 3).

We further analyzed the cumulative effect of these two risk factors for atrophic gastritis and also found a significantly negative effect (*P*_trend_ < 0.001, 95%CI = 0.24–0.32; Table 4).

### Rs895819 genotype and clinical pathological characteristics

To investigate the association of genotype with phenotype, we analyzed the association between rs895819 and the clinical pathological characteristics of gastric cancer patients. No significant association was found between age and sex, macroscopic type, Lauren grade, TNM stage, depth of invasion, lymphatic metastasis and rs895819 genotypes (Supplementary Table 6).

### Association of miR-27a rs895819 with cancer survival prognosis

**Figure 1:**
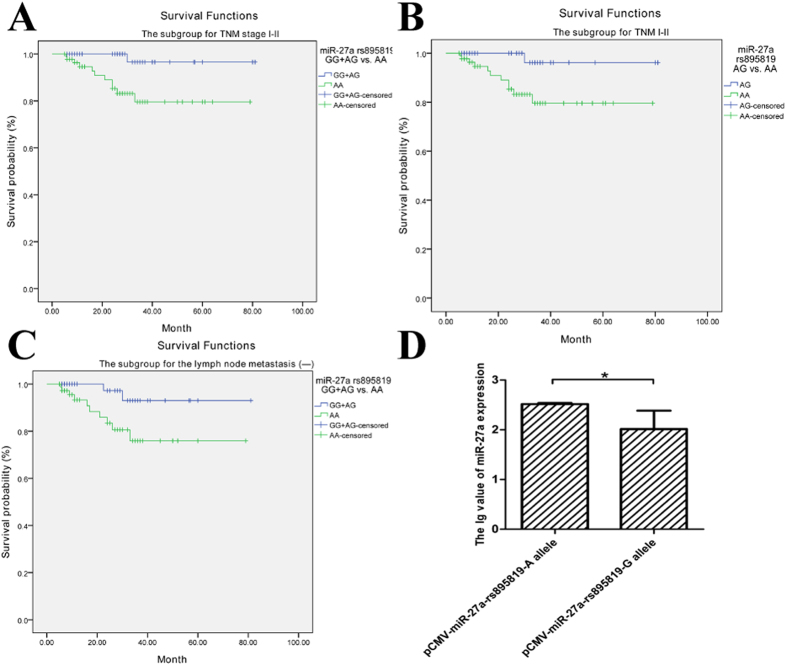
The effect of miR-27a rs895819 A/G SNP on the mature miR-27a expression *in vivo* and *vitro*. (**A**) Kaplan–Meier survival curve analysis with the different genotypes of miR-27a rs895819 (GG + AG vs. AA) for the subgroup for TNM I-II stage. (**B**) Kaplan–Meier survival curve analysis with the different genotypes of miR-27a rs895819 (AG vs. AA) for the subgroup for TNM I-II stage. (**C**) Kaplan–Meier survival curve analysis with the different genotypes of miR-27a rs895819 (GG + AG vs. AA) for the subgroup for lymph node metastasis (−). (**D**) The cell mature miR-27a expression transinfected by different miR-27a rs895819 plasmid. *P = 0.05.

To analyze the relationship between rs895819 and survival time, univariate and multivariate Cox proportional hazard analysis were performed adjusted by adding all SNP variables to the clinicopathological parameters with *P* < 0.05 (TNM stage, lymphatic metastasis and depth of invasion; Supplementary Table 7). No significant difference was discovered between rs895819 and survival, while the result was totally contrary in the subgroups. In the TNM stage I–II subgroup, the overall survival in the subjects carrying with rs895819 AG heterozygosis was better than the AA wild-type (univariate: *P* = 0.036, HR = 0.11; multivariate: *P* = 0.041, HR = 0.12, Fig. 1-A,B). In the lymphatic metastasis subgroup, individuals carrying with rs895819 AG + GG genotype had better survival compared with AA wild-type (univariate: *P* = 0.040, HR = 0.20, Table 5, Fig. 1-C).

### The effect of rs895819 on miR-27a expression

We first analyzed the differential expression of miR-27a between cancer patients and control groups, as well as between tumor tissues and paired non-cancer tissues. miR-27a expression in cancer patients was significantly higher than that of the control groups in serum expression level (*P* < 0.001, Table 6). And the same tendency was revealed in tissue expression level rather than reaching the statistical significance (*P* = 0.063).

To preliminarily explore the effect of rs895819 on mature miR-27a expression, we analyzed the mature miRNA expression based on different SNP genotypes both in tissue and in serum or in cancerous and non-cancerous groups. No significant effect of different rs895819 genotypes on mature miR-27a expression in tissues or in serum was observed (Table 6).

In the *vitro* level, AGS cell line was selected by the screening of miR-27a expression. And the AGS cells were transinfected into two plasmids, pCMV-miR-27a-rs895819-A and pCMV-miR-27a-rs895819-G. After 24 hours, the miR-27a expression level of variant G allele was significantly lower compared with the ancient A allele (*P* = 0.05, Fig. 1-D).

## Discussion

We comprehensively and systematically conducted the risk and survival study for the special miR-27a rs895819 SNP, and further performed the expression study in tissue and in serum or *in vivo* as well as *in vitro*. We found that the rs895819 SNP was associated with an increased risk of atrophic gastritis and gastric cancer. An interaction between rs895819 and *H. pylori* for atrophic gastritis risk was been found. Individuals carrying with the rs895819 AG heterozygosis had better survival in the TNM stage I–II subgroup. And cells carrying with polymorphic-type G allele expressed lower miR-27a level than wild-type A allele.

In this study, we found that rs895819 AG genotype was associated with an increased risk of gastric cancer. It has been reported that rs895819 increased the gastric cancer risk in Chinese population, and could affect transcription proceeding from pri-miRNA to pre-miRNA resulting in the changes in the expression of mature miRNA^2^. It suggested that rs895819 was a functional SNP, and our result was consistent with the previously published study^2^. Only one study has shown negative association of this SNP with atrophic gastritis risk^21^. It has been suggested by Lauren that gastric cancer, especially intestinal-type gastric cancer, develops from atrophic gastritis, which is known to be an important precancerous disease^22^. Our analysis of a second set of samples by MALDI-TOF-MS revealed that the rs895819 SNP was associated with a 1.56-fold increase in the risk of atrophic gastritis, suggesting that this variant is involved in the progression of gastric carcinogenesis, and furthermore, implicating this SNP could be a biomarker of the risk of precancerous atrophic gastritis.

Environmental factors consist of the host’s natural and social environment. The natural environment contains geographical and occupational factors as well as bacterial or viral infection. *H. pylori* infection is an important environmental factor that influences the risk of gastric cancer. In this study, when stratified by the *H. pylori* infection status, the OR value of the *H. pylori*-negative subgroup was found to be higher than that of the overall population (1.62 vs. 1.58, respectively). This suggests that the role of rs895819 SNP will be more apparent when the *H. pylori* factor is removed from the analysis. Further analysis based on this finding showed that rs895819 and *H. pylori* infection status could exert a negative interaction effect. Surprisingly, linear regression analysis showed that accumulation of the risk factors including the rs895819 risk genotype and *H. pylori* infection was associated with a protective effect against carcinogenesis. This accumulation effect revealed by linear regression analysis was consistent with the interaction analysis, which suggested that the association of this rs895819 SNP with *H. pylori* infection was based on antagonism. Both rs895819 SNP and *H. pylori* infection are risk factors for carcinogenesis, as *H. pylori* is a tumour accelerator and the rs895819 SNP is a polymorphism associated with increased gastric cancer risk. However, it can be speculated that rs895819 SNP weakens the carcinogenic role of *H. pylori* in individuals carrying with both two risk factors. Similar situations were observed in the associations of *H. pylori* infection and microsatellite instability with gastric cancer prognosis. A recent meta-analysis of 2,454 gastric cancer patients suggested a protective role for infection in prognosis, and also that *H. pylori*-induced inflammation might modulate antitumor immunity^23^. Microsatellite instability is a hallmark of the DNA-mismatch repair deficiency, which is one of the pathways of gastric carcinogenesis although microsatellite alterations are related to better post-operative survival^24^,^25^. Therefore, it is plausible that the rs895819 SNP has a negative interaction with *H. pylori* infection in gastric carcinogenesis. In our research, we also found that miR-27a rs895819 GG genotype was associated with an increased risk of atrophic gastritis in stage 2. Some scholars have also reported that miRNA expression changes have been already detectable in early stages of gastric carcinogenesis including *H. pylori* induced atrophic gastritis^26^. But whether the variations in miRNAs, especially the SNP in miR-27a, were associated with *H. pylori*-related atrophic gastritis was still not clear. Several investigators were concerned with the association between rs895819 and atrophic gastritis risk, but no significant association^21^,^27^,^28^ was shown. Thus, large-scales and multi-central studies are needed in the near future.

We also found that in the TNM stage I–II or non-lymphatic metastasis subgroups, the patients carrying with *miR-27a* rs895819 AG + GG genotypes had better prognosis. It has been reported that *miR-27a* rs895819 G is associated with better survival^29^, which is consistent with the results of our study. In the risk study, this rs895819 variant genotype showed a risk function, although in the prognosis study, the same variant genotype showed a protective function. These conflicting results are consistent with the observation that the rs895819 SNP weakens the carcinogenic role of *H. pylori*. Several studies have shown that miRNA polymorphism increases cancer risk, but exerts protective function in cancer survival. For example, in one study, *miR-146a* polymorphism was found to have risk function in the non-smoking subgroup of gastric cancer patients, but played a protective role in the intestinal-type subgroup of gastric cancer patients^30^. In another study, *miR-196a2* polymorphism exerted a protective function in head and neck cancer, but was associated with a negative effect on survival in gastric cancer patients^31^. These contradictory findings may result from the different types of cancer, or the association with the effect of miRNA polymorphism on miRNA expression in carcinogenesis.

MiRNA polymorphism could affects miRNA transcription and mature miRNA production^32^. To further explore the possible mechanism of the association between rs895819 and gastric cancer risk as well as prognosis we analyzed mature miR-27a expression in accordance with the polymorphic stratification. We did not found significant statistical effects of the SNP on the mature miR-27a expression both in the serum and in tissue or in cancer and non-cancerous groups. But in the *vivo* experiment, significantly lower mature miR-27a expression level were detected in AGS cells containing pCMV-miR-27a-rs895819-G allele than that of wild-A allele suggesting the rs895819 may effect mature miR-27a expression to a certain extent, which could partly interpret the mechanism involved in the protective function in cancer survival but controversial the increased gastric cancer susceptibility. This phenomenon is very puzzling and interesting. Other scholars also reported controversial results for the miR-27a function, for example, Zhou L *et al*. found that miR-27a could promote cancer cell proliferation^33^; while Wang X *et al*. reported that miR-27a acted as a tumor suppressor by suppressing oncogene MAP2K4 *in vivo* experiment^34^. Accordingly, some thought it was an “oncogene”^33^,^35^ but some believed it could induce the apoptosis^36^. Thus, it is still unclear whether the mature miR-27a acts as an “oncogene” or “tumor suppressor” during carcinogenesis. Maybe miR-27a play a role as double-edged sword, in that way, it is also still unclear that when miR-27a acts as an “oncogene” or “tumor suppressor”. The real function of miR-27a still needs to be investigated in the future.

In 2012, Yang reported that rs895819 polymorphism is associated with an unusual structure making primer extension possible from only one direction (not overlapping with any SNP). The use of direct sequencing or MALDI-TOF Mass-ARRAY is essential for *miR-27a* rs895819 and rs11671784 genotyping because qPCR and PCR-RFLP are associated with unacceptable risk of false-positive genotyping^14^. Later in 2013, other researchers noted that only the direct sequencing or MALDI-TOF Mass-ARRAY was acceptable for genotyping this rs895819 polymorphism^37^. Subsequently, however, the special structure of rs895819 was rarely considered and Taqman or PCR-RFLP technologies were still used, rendering the association of this polymorphism and disease risk being inconsistent. To make the unusual structure of this rs895819 polymorphism being compatible, we used direct sequencing and Mass-ARRAY technology to increase the credibility of our study. Furthermore, we found only one case of the rs11671784 AG genotype among 724 samples was analyzed by direct sequencing. This distribution frequency is in accordance with the NCBI databases (http://www.ncbi.nlm.nih.gov/projects/SNP/snp_ref.cgi?rs=11671784). Several studies^2^,^38^ investigated this rs11671784 polymorphism using PCR-RFLP or Taqman technology and the distribution frequency of the variant allele was higher than 50% both in the control and cancerous groups, which increases the risk of false genotyping.

There are some limitations of our study. First, the small sample size limited the subgroup analysis of rare genotypes. Second, complete information for other environmental factors is required to analyze polymorphism interaction effects.

In summary, we found that the rs895819 variant genotype was associated with an increased risk of atrophic gastritis and gastric cancer. In the stratified analysis, these effects were more significant in the female, age >60, *H. pylori-*negative and non-drinkers subgroups. The negative interaction between rs895819 and *H. pylori* in atrophic gastritis risk should be noted. For the survival analysis, individuals carrying with the rs895819 AG heterozygosis had better survival compared with individuals with the AA wild-type genotype in the TNM stage I–II subgroup. And it was confirmed by the *vitro* study that cells carrying polymorphic-type G allele expressed lower miR-27a than wild-type A allele. In conclusion, *miR-27a* rs895819 is implicated as a biomarker for gastric cancer and atrophic gastritis risk, and interacts with *H. pylori* in gastric carcinogenesis.

## Methods

### Study design

This study was approved by the Ethics Committee of the First Affiliated Hospital of China Medical University and the methods were carried out in accordance with the relevant guidelines and regulations. All participants were non-consanguineous ethnic Han Chinese. This study was divided into three parts focusing on risk, prognosis and miRNA expression. For the evaluation of risk, the study design comprised two stages using two different detection methods (direct sequencing and Sequenom Mass-ARRAY analysis) to investigate a total of 939 gastric cancer patients, 1,067 atrophic gastritis patients and 1,166 controls. To explore the association between *miR-27a* rs895819 with the risk of gastric cancer and atrophic gastritis, the first stage of the study involved direct sequencing of the samples from 724 retrospectively recruited individuals, consisting of 215 gastric cancer patients, 205 atrophic gastritis cases and 304 controls who attended the First Affiliated Hospital of China Medical University between 2005 and 2010. In the second stage of the study, the Sequenom Mass-ARRAY platform was used to investigate a total of 2,448 cases, consisting of 724 gastric cancer cases, 862 atrophic gastritis cases and 862 matched healthy controls from the Zhuanghe Gastric Diseases Screening Program^39^ or from patients who attended the First Affiliated Hospital of China Medical University between 2002 and 2013. All the subjects in this study were endoscopically and histologically confirmed. The classification of gastric cancer was based on Lauren’s classification^22^, which was divided into intestinal-type and diffuse-type for subgroup analysis. The classification and grading of gastritis were based on the Updated Sydney System^40^,^41^. Subjects who were endoscopically and histologically confirmed with normal mucosa or only minimal gastritis without other systemic disease and stomach diseases served as controls. Written informed consent was obtained from all participants. Medical histories (including details of age, sex, smoking, and alcohol consumption) were obtained by questionnaire and the records were computerized.

To further investigate the correlation of risk-associated polymorphisms with clinicopathologic parameters and survival in gastric cancer patients, we analyzed the data of 357 gastric cancer cases, which were all suffered surgical resection and the information of death or survival was available. The tumour histological grade was evaluated according to the World Health Organization criteria and tumours were staged using the 7^th^ edition of the TNM Staging System of the International Union Against Cancer (UICC)/American Joint Committee on Cancer (AJCC) (2010) based on post-operative pathologic examinations. Patients (i) with distant metastasis found preoperatively, (ii) who underwent preoperative radiotherapy or chemotherapy, or (iii) with incomplete pathological data entries were excluded from the survival analysis. The information on clinicopathologic parameters were collected at baseline and the complete pathological data were obtained including macroscopic type, histologic grade, depth of invasion, number of LNs retrieved, number of metastatic LNs, and number of tumor deposits retrieved. The following data were obtained for all patients: date of death (if applicable), cause of death (if applicable), and date of follow-up. The primary endpoint was cause-specific survival duration from the date of gastric cancer diagnosis to the date of death. The median follow-up time was 23.0 months.

For the evaluation of a correlation between rs895819 and expression of the corresponding mature miRNA in serum, 170 cases consisting of 87 gastric cancer patients and 83 healthy controls were examined. In addition, for the assessment of correlation between rs895819 and miR-27a expression in gastric tissue, 32 non-cancerous specimens and 31 gastric cancerous specimens were obtained from 35 patients who underwent gastrectomy at the First Affiliated Hospital of China Medical University between 2009 and 2013.

### Genotyping of subjects

Genomic DNA was extracted as described previously^42^, with some modifications. The genotyping in stage 1 was performed by direct sequencing carried out by Huada Gene Company (Shenzhen, China) to identify rs895819 and rs11671784 genotypes. The fragments for sequencing were amplified by PCR using the primers F: 5′-AACTTAGCCACTGTGAACACG-3′ and R: 5′-AGTTGCTGTAGCCTCCTTGTC-3′ with an annealing temperature of 59 °C. The genotyping in stage 2 assay was performed using the Sequenom Mass-ARRAY platform (Sequenom, San Diego, CA, USA) by Biomiao (Beijing, China) as described previously^43^. 5% samples in the same groups were random chosen to be detected in both the direct sequencing and MALDI-TOF Mass-ARRAY methods to test the consistency of these two methods, with a concordance rate of 100%. Another 5% samples were random chosen to be detected in the same method, and the consistency also reached 100%.

### Serum H. pylori-IgG titer determination

Serum *H. pylori*-IgG titers were determined by enzyme linked immunosorbent assay (ELISA, *Helicobacter pylori*-IgG kit; Biohit, Helsinki, Finland) according to a previously described method^44^. *H. pylori*-IgG titers >35 was judged to be positive.

### RNA Extraction and real-time PCR analysis of miRNA expression *in vivo*

MiRNA was extracted from serum and tissue samples as described previously^45^ with some modifications. The One Step Prime Script miRNA cDNA (Perfect Real-Time) Kit (TAKARA Biotechnology Co., Ltd, Dalian, China) was used to generate cDNA and the miRcute miRNA qPCR detection kit (SYBR) (TIANGEN Biotech Co., Ltd, Beijing, China) was used for real-time PCR analysis with the primer 5′-CGCGTTCACCGTGGCTAAGTTCC-3′. The methods used have been described previously^46^,^47^.

### Transient transfection and Real-time PCR reaction for miRNA expression *in vitro*

The commercial expression plasmid pCMV-miR-27a-rs895819-A was purchased from Genechem Company (Genechem Biotech Co., Ltd, Shanghai, China). And the polymorphic plasmid was conducted for site-specific mutagenesis from A to G (pCMV-miR-27a-rs895819-G) by Genechem Company and confirmed by sequencing. Then, the lowest miR-27a expression cell line, AGS, was selected for transfection (More details see Supplementary Methods and Supplementary Figure 1). After 24 hours, the total RNA of cells was extracted and Real-time PCR was used to detect miR-27a expression after reverse transcription in order to compare the mature miR-27a produced by pCMV-miR-27a-rs895819-G vs. pCMV-miR-27a-rs895819-A.

### Statistical analysis

To determine the genetic models for rs895819 SNP, we employed the method suggested by Thakkinstian *et al*.^20^. The strength of association between genotype and disease risk was assessed by odds ratio (OR) and 95% confidence interval (CI) measures, adjusted by sex, age and status of *H. pylori* infection. Genetic effects for three pairwise comparisons were calculated to determine the optimal genetic model for each polymorphism, including OR1 for GG versus AA, OR2 for AG versus AA, and OR3 for GG versus AG. If OR1 = OR3 ≠ 1 and OR2 = 1, a recessive model is suggested and GG was compared with the group of AG plus AA. If OR1 = OR2 ≠ 1 and OR3 = 1, a dominant model is indicated and the group of GG plus AG was compared with AA. If OR2 = 1/OR3 ≠ 1 and OR1 = 1, a complete overdominant model is suggested and AG was compared with the group of GG and AA. If OR1 > OR2 > 1 and OR1 > OR3 > 1, or OR1 < OR2 < 1 and OR1 < OR3 < 1, a codominant model is indicated and GG was compared with AG and with AA. Hardy–Weinberg equilibrium (HWE) among the controls was analyzed using the *χ*^2^ test. Continuous variables were represented as mean ± standard deviation (SD) and compared by analysis of variance (ANOVA), while the discrete variables were represented as frequencies and percentages and compared by the *χ*^2^ test. Multivariate logistic regression with adjustment for age, sex and *H. pylori* infection was used to assess the association between *miR-27a* polymorphism and gastric disease risk. The heterogeneity test for the gender, age and *H. pylori* infection status was performed by STATA software, version 11.0 (STATA Corp., College Station, TX, USA). Data for smoking and alcohol consumption was missing in nearly a third of cases and was not suitable for being adjustment factors. Therefore, these characteristics were used only as stratified factors for analysis of the association between the *miR-27a* polymorphism and disease risks^48^. Univariate and multivariate survival analyses were carried out using the log-rank test and the Cox proportional hazards model. The survival curves were mapped using the Kaplan–Meier method. Multivariate survival analysis was carried out by adding details of the SNP to all clinicopathological parameters with *P* < 0.05. In addition, the lg value of the copies of miRNA was used for a normal distribution, and the effect of *miR-27a* polymorphism on expression level was tested by ANOVA. The lg value of the miR-27a copies expressed by the AGS cells carrying with different miR-27a alleles were analyzed by nonparametric test because of the abnormal distribution. Statistical analysis was performed using SPSS version 18.0 software (SPSS, Chicago, IL, USA) and *P-*values < 0.05 was considered to be of statistical significance.

## Additional Information

**How to cite this article**: Xu, Q. *et al. MiR-27a* rs895819 is involved in increased atrophic gastritis risk, improved gastric cancer prognosis and negative interaction with *Helicobacter pylori. Sci. Rep.* **7**, 41307; doi: 10.1038/srep41307 (2017).

**Publisher's note:** Springer Nature remains neutral with regard to jurisdictional claims in published maps and institutional affiliations.

## Supplementary Material

Supplementary Dataset 1

## Figures and Tables

**Table 1:** The association of miR-27a rs895819 polymorphisms and gastric cancer/atrophic gastritis risk^a^.

Genotype	NCBI Ref (%)	CON (%)	AG (%)	AG vs CON		CON (%)	GC (%)	GC vs CON	
				OR (95% CI)	*P*			OR (95% CI)	*P*
**Stage 1**		**n = 304**	**n = 205**			**n = 304**	**n = 215**		
AA	21 (48.8)	171 (56.3)	110 (53.7)	1 (Ref)		171 (56.3)	111 (51.6)	1 (Ref)	
AG	17 (39.5)	111 (36.5)	83 (40.5)	1.15 (0.79–1.68)	0.463	111 (36.5)	97 (45.1)	1.32 (0.91–1.91)	0.141
GG	5 (11.6)	22 (7.2)	12 (5.9)	0.84 (0.39–1.79)	0.646	22 (7.2)	7 (3.3)	0.47 (0.19–1.14)	0.094
GG + AG VS. AA				1.10 (0.77–1.58)	0.559			1.17 (0.82–1.67)	0.386
GG VS. AG + AA				0.80 (0.38–1.68)	0.559			**0**.**41** (**0**.**17**–**0**.**99**)	**0**.**047**
*P*_HWE_^b^	0.590	0.498							
**Stage 2**		**n = 862**	**n = 862**			**n = 729**	**n = 724**		
AA		482 (55.9)	467 (54.2)	1 (Ref)		411 (56.4)	369 (51.0)	1 (Ref)	
AG		340 (39.4)	336 (39.0)	0.98 (0.80–1.21)	0.874	282 (38.7)	311 (43.0)	1.21 (0.97–1.51)	0.089
GG		40 (4.6)	59 (6.8)	**1**.**58** (**1**.**01**–**2**.**46**)	**0**.**045**	36 (4.9)	44 (6.1)	1.35 (0.84–2.18)	0.220
GG + AG VS. AA				1.04 (0.85–1.27)	0.684			1.23 (0.99–1.52)	0.061
GG VS. AG + AA				**1**.**56** (**1**.**01**–**2**.**41**)	**0**.**045**			1.22 (0.76–1.94)	0.415
**Meta-analysis**		**n = 1166**	**n = 1067**			**n = 1033**	**n = 939**		
AA		653 (56.0)	577 (54.1)	1 (Ref)		582 (56.3)	480 (51.1)	1 (Ref)	
AG		451 (38.7)	419 (39.3)	1.02 (0.85–1.23)	0.797	393 (38.0)	408 (43.5)	**1**.**24** (**1**.**03**–**1**.**50**)	**0**.**027**
GG		62 (5.3)	71 (6.7)	1.30 (0.89–1.88)	0.174	58 (5.6)	51 (5.4)	1.03 (0.68–1.54)	0.906
GG + AG VS. AA				1.28 (0.89–1.84)	0.190			**1**.**21** (**1**.**01**–**1**.**45**)	**0**.**043**
GG VS. AG + AA				1.06 (0.89–1.26)	0.537			0.92 (0.62–1.37)	0.681
GG vs AA (OR1)				1.30 (0.89–1.88)	0.174			1.03 (0.68–1.54)	0.906
AG vs. AA (OR2)				1.02 (0.85–1.23)	0.797			**1.24 (1.03**–**1.50)**	**0.027**
GG vs AG (OR3)				1.25 (0.86–1.83)	0.248			0.81 (0.54–1.22)	0.318
Codominant model^c^				1.06 (0.89–1.26)	0.537				
Complete overdominant model^d^								**1.24 (1.03**–**1.49)**	**0.025**

Note: ^a^Using Logistic Regession adjusted by sex, age and *H. pylori* infection; ^b^Means Hardy–Weinberg Equilibrium in population; ^c^A codominant model was indicated for rs895819 in the comparison of atrophic gastritis vs. control group, and GG was compared with AG and with AA. ^d^A complete overdominant model was implied for rs895819 in the comparison of gastric cancer vs. control group. The original grouping was collapsed and the new group of AG heterozygote was compared with GG and with AA. CON: controls; AG: atrophic gastritis; GC: gastric cancer; NCBI Ref: the reference frequencies of these polymorphisms in Beijing Han, China in NCBI database.

**Table 2:** Association of miR-27a rs895819 polymorphism with the risk of atrophy gastritis and gastric cancer stratified by host characteristics.

Variables	Genotype	AG vs CON	OR (95% CI)	*P*	GC vs CON	OR (95% CI)	*P*
Gender^a^		n = 1067 vs 1166			n = 939 vs 1033		
Male	AA	343/377	1 (Ref)		334/377	1 (Ref)	
	AG	238/255	0.97 (0.76–1.23)	0.803	268/255	1.15 (0.92–1.45)	0.227
	GG	33/38	0.93 (0.56–1.55)	0.787	33/38	0.96 (0.58–1.58)	0.875
	AG + GG VS. AA		1.00 (0.77–1.22)	0.772		1.13 (0.90–1.41)	0.282
	GG VS. AG + AA		0.95 (0.57–1.56)	0.829		0.89 (0.55–1.46)	0.648
Female	AA	234/276	1 (Ref)		146/205	1 (Ref)	
	AG	181/196	1.12 (0.84–1.47)	0.443	140/138	**1**.**44** (**1**.**03**–**2**.**00**)	**0**.**032**
	GG	38/24	**1**.**86** (**1**.**06**–**3**.**27**)	**0**.**030**	18/20	1.17 (0.58–2.36)	0.661
	AG + GG VS. AA		1.20 (0.92–1.56)	0.183		**1**.**39** (**1**.**01**–**1**.**91**)	**0**.**045**
	GG VS. AG + AA		**1**.**77** (**1**.**03**–**3**.**06**)	**0**.**040**		0.98 (0.50–1.95)	0.959
Age^a^		n = 1067 vs 1166			n = 939 vs 1033		
≤60	AA	406/477	1 (Ref)		334/406	1 (Ref)	
	AG	283/324	1.02 (0.82–1.26)	0.886	232/266	1.06 (0.84–1.33)	0.655
	GG	46/39	1.39 (0.87–2.21)	0.168	32/35	1.02 (0.61–1.70)	0.948
	AG + GG VS. AA		1.06 (0.86–1.30)	0.609		1.05 (0.84–1.32)	0.667
	GG VS. AG + AA		1.37 (0.87–2.16)	0.173		1.01 (0.61–1.66)	0.983
>60	AA	171/176	1 (Ref)		146/176	1 (Ref)	
	AG	136/127	1.07 (0.76–1.49)	0.712	176/127	**1**.**74** (**1**.**25**–**2**.**42**)	**0**.**001**
	GG	25/23	1.14 (0.61–2.14)	0.682	19/23	1.10 (0.56–2.14)	0.785
	AG + GG VS. AA		1.08 (0.78–1.48)	0.659		**1**.**63** (**1**.**18**–**2**.**24**)	**0**.**003**
	GG VS. AG + AA		1.11 (0.60–2.07)	0.731		0.85 (0.44–1.62)	0.614
*H. pylori*^*b*^		n = 1067 vs 1166			n = 939 vs 1033		
negative	AA	250/469	1 (Ref)		240/165	1 (Ref)	
	AG	179/315	1.06 (0.84–1.35)	0.629	204/117	1.27 (1.00–1.62)	0.055
	GG	36/41	**1**.**62** (**1**.**01**–**2**.**60**)	**0**.**047**	27/19	1.08 (0.63–1.85)	0.782
	AG + GG VS. AA		1.12 (0.89–1.41)	0.319		1.24 (0.98–1.57)	0.069
	GG VS. AG + AA		1.58 (0.99–2.51)	0.054		0.94 (0.55–1.58)	0.803
positive	AA	327/184	1 (Ref)		240/417	1 (Ref)	
	AG	240/136	0.98 (0.74–1.30)	0.899	204/276	1.19 (0.88–1.61)	0.262
	GG	35/21	0.92 (0.52–1.64)	0.785	24/39	0.98 (0.52–1.82)	0.936
	AG + GG VS. AA		0.97 (0.74–1.27)	0.820		1.15 (0.86–1.55)	0.335
	GG VS. AG + AA		0.93 (0.53–1.64)	0.810		0.90 (0.49–1.64)	0.720
Smoking^b^		n = 548 vs 586			n = 333 vs 500		
Never smoker	AA	204/224	1 (Ref)		102/180	1 (Ref)	
	AG	143/142	1.08 (0.78–1.50)	0.642	69/105	1.17 (0.78–1.77)	0.444
	GG	26/18	1.95 (0.99–3.85)	0.055	14/16	1.64 (0.74–3.67)	0.225
	AG + GG VS. AA		1.17 (0.85–1.60)	0.330		1.23 (0.83–1.82)	0.295
	GG VS. AG + AA		1.87 (0.96–3.64)	0.066		1.53 (0.70–3.34)	0.288
Ever smoker	AA	94/109	1 (Ref)		74/107	1 (Ref)	
	AG	68/82	0.95 (0.60–1.50)	0.828	68/81	1.13 (0.71–1.82)	0.606
	GG	11/11	1.21 (0.48–3.09)	0.685	6/11	0.60 (0.20–1.84)	0.376
	AG + GG VS. AA		0.98 (0.64–1.52)	0.936		1.06 (0.67–1.67)	0.819
	GG VS. AG + AA		1.23 (0.50–3.07)	0.653		0.54 (0.18–1.64)	0.277
Alcohol drinking^b^		n = 547 vs 585			n = 296 vs 499		
Nondrinker	AA	239/256	1 (Ref)		105/211	1 (Ref)	
	AG	157/165	1.07 (0.78–1.46)	0.687	71/127	1.10 (0.74–1.64)	0.641
	GG	27/17	**2**.**08** (**1**.**04**–**4**.**16**)	**0**.**037**	13/15	1.43 (0.62–3.33)	0.406
	AG + GG VS. AA		1.15 (0.85–1.55)	0.358		1.13 (0.77–1.66)	0.521
	GG VS. AG + AA		**1**.**99** (**1**.**01**–**3**.**90**)	**0**.**047**		1.35 (0.59–3.08)	0.484
Drinker	AA	60/76	1 (Ref)		52/75	1 (Ref)	
	AG	53/60	0.99 (0.58–1.67)	0.959	50/60	1.20 (0.70–2.06)	0.503
	GG	11/11	1.22 (0.49–3.05)	0.677	5/11	0.73 (0.23–2.30)	0.587
	AG + GG VS. AA		1.03 (0.63–1.70)	0.902		1.14 (0.68–1.93)	0.620
	GG VS. AG + AA		1.33 (0.54–3.27)	0.542		0.71 (0.23–2.21)	0.555

Note: ^a^Using Logistic Regession adusted by the other two factors of gender, age and *H. pylori* infection status. ^b^Using Logistic Regession adusted by gender, age and *H. pylori* infection status. CON:controls; AG: atrophic gastritis; GC:gastric cancer.

**Table 3:** The interaction of *miR-27a* rs895819 polymorphism and *H. pylori* infection statue in the risk of gastric cancer/atrophic gastritis^a^.

Genotype		AG vs CON		GC vs CON	
		*H. pylori*		*H. pylori*	
		(−)	(+)	(−)	(+)
AG + AA	Case/Control	385/709	528/284	395/618	396/246
	OR (95%CI)	1 (Ref)	3.44 (2.84–4.16)	1 (Ref)	2.53 (2.07–3.10)
GG	Case/Control	80/116	74/57	73/114	75/55
	OR (95%CI)	1.27 (0.93–1.73)	2.38 (1.65–3.44)	1.00 (0.73–1.38)	2.13 (1.47–3.08)
		***P***_***interaction***_** = 0**.**013**		*P*_*interaction*_ = 0.505	
		**OR** (**95%CI**)** = 0**.**54** (**0**.**33**–**0**.**88**)		OR (95%CI) = 0.84 (0.51–1.39)	

Note: ^a^*P* for interaction was used Logistic Regression adjusted by gender and age. CON: controls; AG: atrophic gastritis; GC: gastric cancer.

**Table 4:** Cumulative effect of the interacting risk factors of *miR-27a* rs895819-*H. pylori* infection on the atrophic gastritis risk.

No. of interacting risk factors	Total population		
	Cases/controls	*P* ^a^	OR (95% CI)
0	329/592		1 (ref)
1	502/259	<0.001	3.53 (2.89–4.33)
2	31/11	<0.001	5.06 (2.51–10.21)
		***P*** **trend <0**.**001**, **95%CI = 0**.**24**–**0**.**32**	

Note: ^a^Adjusted by sex and age.

**Table 5:** Univariate and multivariate cox proportional hazard analysis for *miR-27a* rs895819 polymorphism.

Genotype	All GC	Death	MST^a^ (M)	Univariate		Multivariate^c^	
				HR (95% CI)	*P*	HR (95% CI)	*P*
	n = 357	n = 89					
AA	184	48	58.4^b^	1 (Ref)		1 (Ref)	
AG	148	36	58.8^b^	0.89 (0.58–1.37)	0.597	0.84 (0.54–1.29)	0.415
GG	25	5	45.4^b^	0.75 (0.30–1.88)	0.535	0.62 (0.25–1.57)	0.316
AG + GG VS. AA				0.87 (0.57–1.32)	0.510	0.80 (0.53–1.21)	0.294
GG VS. AG + AA				0.80 (0.32–1.97)	0.625	0.68 (0.27–1.67)	0.398
Subgroup analysis for TNM stage I–II							
I–II	n = 171	n = 12					
AA	91	11	67.0^b^	1 (Ref)		1 (Ref)	
AG	68	1	79.0^b^	**0**.**11** (**0**.**01**–**0**.**87**)	**0**.**036**	**0**.**12** (**0**.**02**–**0**.**91**)	**0**.**041**
GG	12	0	NA	NA	NA	NA	NA
AG + GG VS. AA				**0**.**10** (**0**.**01**–**0**.**77**)	**0**.**027**	**0**.**10** (**0**.**01**–**0**.**80**)	**0**.**030**
GG VS. AG + AA				NA	NA	NA	NA
Subgroup analysis for Lymphatic metastasis (−)							
Negative	n = 141	n = 12					
AA	74	10	64.7^b^	1 (Ref)		1 (Ref)	
AG	58	2	76.9^b^	0.22 (0.05–1.02)	0.054	0.25 (0.06–1.15)	0.076
GG	9	0	NA	NA	NA	NA	NA
AG + GG VS. AA				**0**.**20** (**0**.**05**–**0**.**93**)	**0**.**040**	0.2 (0.05–1.03)	0.054
GG VS. AG + AA				NA	NA	NA	NA

Note: HR, hazard ratio; CI, confidence interval; ^a^MST, median survival time (months). ^b^when MST could not be calculated, mean survival time was provided. ^c^Multivariate survival analysis was carried out by adding all the SNP variables to the clinicopathological parameters with *P* < 0.05 including TNM stage, lymphatic metastasis and depth of invasion. NA, not available.

**Table 6:** The effect of miR-27a rs895819 polymorphism to its mature miR-27a expression.

	Serum expression					Tissue expression *in situ*				
	Cancer		Controls			Cancer Tissue		Noncancer Tissues		
	n	Mean ± SD	n		Mean ± SD	n	Mean ± SD		n	Mean ± SD
Total	87	2.12 ± 1.14	83		1.46 ± 0.73	31	8.71 ± 0.37	32		8.55 ± 0.31
*P*^a^	**<0**.**001**						0.063			
Genotype										
AA	38	2.25 ± 1.13		44	1.57 ± 0.78	14	8.84 ± 0.40		16	8.53 ± 0.32
AG	47	2.01 ± 1.14		34	1.33 ± 0.71	16	8.64 ± 0.30		16	8.56 ± 0.30
GG	2	2.02 ± 1.90		5	1.35 ± 0.18	1	8.18		0	NA
*P*^b^		0.630			0.340		0.103			0.744

Note: ^a^Means the dependent sample-t test was used to calculate the difference betweenthe expressions of tumor and controls; ^b^Means the variance analysis was used to calculate the difference between the expressions of three genotypes.

## References

[b1] Shastry B. S. SNPs: impact on gene function and phenotype. Methods in molecular biology (Clifton, NJ, 578, 3–22 (2009). 19768584 10.1007/978-1-60327-411-1_1

[b2] Song B., Yan G., Hao H. & Yang B. rs11671784 G/A and rs895819 A/G polymorphisms inversely affect gastric cancer susceptibility and miR-27a expression in a Chinese population. Medical science monitor: international medical journal of experimental and clinical research 20, 2318–2326 (2014). 25399405 10.12659/MSM.892499 PMC4245106

[b3] Pivarcsi A., Stahle M. & Sonkoly E. Genetic polymorphisms altering microRNA activity in psoriasis–a key to solve the puzzle of missing heritability? Experimental dermatology 23, 620–624 (2014). 24917490 10.1111/exd.12469

[b4] Ha M. & Kim V. N. Regulation of microRNA biogenesis. Nature reviews Molecular cell biology 15, 509–524 (2014). 25027649 10.1038/nrm3838

[b5] Xu Q., He C. Y., Liu J. W. & Yuan Y. Pre-miR-27a rs895819A/G polymorphisms in cancer: a meta-analysis. PLoS One 8, e65208 (2013). 23762318 10.1371/journal.pone.0065208 PMC3676439

[b6] Zeng Y., Yi R. & Cullen B. R. Recognition and cleavage of primary microRNA precursors by the nuclear processing enzyme Drosha. The EMBO journal 24, 138–148 (2005). 15565168 10.1038/sj.emboj.7600491 PMC544904

[b7] Yang R. . A genetic variant in the pre-miR-27a oncogene is associated with a reduced familial breast cancer risk. Breast cancer research and treatment 121, 693–702 (2010). 19921425 10.1007/s10549-009-0633-5

[b8] Kontorovich T. . Single nucleotide polymorphisms in miRNA binding sites and miRNA genes as breast/ovarian cancer risk modifiers in Jewish high-risk women. International journal of cancer 127, 589–597 (2010). 19950226 10.1002/ijc.25065

[b9] Shi D. . A genetic variant in pre-miR-27a is associated with a reduced renal cell cancer risk in a Chinese population. PLoS One 7, e46566 (2012). 23118855 10.1371/journal.pone.0046566 PMC3484143

[b10] Sun Q. . Hsa-mir-27a genetic variant contributes to gastric cancer susceptibility through affecting miR-27a and target gene expression. Cancer science 101, 2241–2247 (2010). 20666778 10.1111/j.1349-7006.2010.01667.x PMC11159034

[b11] Zhang M. . Associations of miRNA polymorphisms and female physiological characteristics with breast cancer risk in Chinese population. European journal of cancer care 21, 274–280 (2012). 22074121 10.1111/j.1365-2354.2011.01308.x

[b12] Hezova R. . Evaluation of SNPs in miR-196-a2, miR-27a and miR-146a as risk factors of colorectal cancer. World journal of gastroenterology: WJG 18, 2827–2831 (2012). 22719192 10.3748/wjg.v18.i22.2827 PMC3374987

[b13] Catucci I. . The SNP rs895819 in miR-27a is not associated with familial breast cancer risk in Italians. Breast cancer research and treatment 133, 805–807 (2012). 22415478 10.1007/s10549-012-2011-y

[b14] Yang R. & Burwinkel B. A bias in genotyping the miR-27a rs895819 and rs11671784 variants. Breast cancer research and treatment 134, 899–901 (2012). 22752288 10.1007/s10549-012-2140-3

[b15] de Martel C., Forman D. & Plummer M. Gastric cancer: epidemiology and risk factors. Gastroenterology clinics of North America 42, 219–240 (2013). 23639638 10.1016/j.gtc.2013.01.003

[b16] Meng W. . Role of Helicobacter pylori in gastric cancer: advances and controversies. Discovery medicine 20, 285–293 (2015). 26645900

[b17] Hong J. B., Zuo W., Wang A. J. & Lu N. H. Helicobacter pylori Infection Synergistic with IL-1beta Gene Polymorphisms Potentially Contributes to the Carcinogenesis of Gastric Cancer. International journal of medical sciences 13, 298–303 (2016). 27076787 10.7150/ijms.14239 PMC4829543

[b18] Zhang R. G., Duan G. C., Fan Q. T. & Chen S. Y. Role of Helicobacter pylori infection in pathogenesis of gastric carcinoma. World journal of gastrointestinal pathophysiology 7, 97–107 (2016). 26909232 10.4291/wjgp.v7.i1.97 PMC4753193

[b19] Figura N., Marano L., Moretti E. & Ponzetto A. Helicobacter pylori infection and gastric carcinoma: Not all the strains and patients are alike. World journal of gastrointestinal oncology 8, 40–54 (2016). 26798436 10.4251/wjgo.v8.i1.40 PMC4714145

[b20] Thakkinstian A. . A method for meta-analysis of molecular association studies. Statistics in medicine 24, 1291–1306 (2005). 15568190 10.1002/sim.2010

[b21] Song M. Y. . Genetic polymorphisms of miR-146a and miR-27a, H. pylori infection, and risk of gastric lesions in a Chinese population. PLoS One 8, e61250 (2013). 23613822 10.1371/journal.pone.0061250 PMC3629121

[b22] Lauren P. The Two Histological Main Types of Gastric Carcinoma: Diffuse and So-Called Intestinal-Type Carcinoma. an Attempt at a Histo-Clinical Classification. Acta pathologica et microbiologica Scandinavica 64, 31–49 (1965). 14320675 10.1111/apm.1965.64.1.31

[b23] Wang F. . Protective role of Helicobacter pylori infection in prognosis of gastric cancer: evidence from 2,454 patients with gastric cancer. PLoS One 8, e62440 (2013). 23667477 10.1371/journal.pone.0062440 PMC3646839

[b24] Wu M. S. . Alterations of BAT-26 identify a subset of gastric cancer with distinct clinicopathologic features and better postoperative prognosis. Hepato-gastroenterology 49, 285–289 (2002). 11941977

[b25] Lee H. S. . Distinct clinical features and outcomes of gastric cancers with microsatellite instability. Modern pathology: an official journal of the United States and Canadian Academy of Pathology, Inc. 15, 632–640 (2002). 10.1038/modpathol.3880578 12065777

[b26] Link A., Kupcinskas J., Wex T. & Malfertheiner P. Macro-role of microRNA in gastric cancer. Digestive diseases (Basel, Switzerland) 30, 255–267 (2012). 22722550 10.1159/000336919

[b27] Arisawa T. . A polymorphism of microRNA 27a genome region is associated with the development of gastric mucosal atrophy in Japanese male subjects. Digestive diseases and sciences 52, 1691–1697 (2007). 17546506 10.1007/s10620-006-9648-5

[b28] Kupcinskas J. . Gene polymorphisms of micrornas in Helicobacter pylori-induced high risk atrophic gastritis and gastric cancer. PLoS One 9, e87467 (2014). 24475294 10.1371/journal.pone.0087467 PMC3903675

[b29] Amstutz U. . Polymorphisms in MIR27A Associated with Early-Onset Toxicity in Fluoropyrimidine-Based Chemotherapy. Clin Cancer Res. 21, 2038–2044 (2015). 25655103 10.1158/1078-0432.CCR-14-2817

[b30] Ahn D. H. . Association of the miR-146aC > G, miR-149T > C, miR-196a2T > C, and miR-499A > G polymorphisms with gastric cancer risk and survival in the Korean population. Molecular carcinogenesis (2012). 10.1002/mc.21962 23001871

[b31] Christensen B. C. . Mature microRNA sequence polymorphism in MIR196A2 is associated with risk and prognosis of head and neck cancer. Clin Cancer Res. 16, 3713–3720 (2010). 20501619 10.1158/1078-0432.CCR-10-0657 PMC2914465

[b32] Calin G. A. . A MicroRNA signature associated with prognosis and progression in chronic lymphocytic leukemia. The New England journal of medicine 353, 1793–1801 (2005). 16251535 10.1056/NEJMoa050995

[b33] Liu T. . MicroRNA-27a functions as an oncogene in gastric adenocarcinoma by targeting prohibitin. Cancer letters 273, 233–242 (2009). 18789835 10.1016/j.canlet.2008.08.003

[b34] Wan X. . Androgen-induced miR-27A acted as a tumor suppressor by targeting MAP2K4 and mediated prostate cancer progression. The international journal of biochemistry & cell biology 79, 249–260 (2016). 27594411 10.1016/j.biocel.2016.08.043

[b35] Zhou L. . MiR-27a-3p functions as an oncogene in gastric cancer by targeting BTG2. Oncotarget (2016). 10.18632/oncotarget.10460 PMC5239526 27409164

[b36] Nie M. . miR-23a and miR-27a promote human granulosa cell apoptosis by targeting SMAD5. Biology of reproduction 93, 98 (2015). 26400397 10.1095/biolreprod.115.130690

[b37] Zhang N. . A genetic variant in pre-miR-27a is associated with a reduced breast cancer risk in younger Chinese population. Gene 529, 125–130 (2013). 23954879 10.1016/j.gene.2013.07.041

[b38] Yang Q. . Genetic variations in miR-27a gene decrease mature miR-27a level and reduce gastric cancer susceptibility. Oncogene 33, 193–202 (2014). 23246964 10.1038/onc.2012.569

[b39] Tu H. . Temporal changes in serum biomarkers and risk for progression of gastric precancerous lesions: a longitudinal study. International journal of cancer 136, 425–434 (2015). 24895149 10.1002/ijc.29005 PMC4354768

[b40] Dixon M. F., Genta R. M., Yardley J. H. & Correa P. Classification and grading of gastritis. The updated Sydney System. International Workshop on the Histopathology of Gastritis, Houston 1994. The American journal of surgical pathology 20, 1161–1181 (1996). 8827022 10.1097/00000478-199610000-00001

[b41] Stolte M. & Meining A. The updated Sydney system: classification and grading of gastritis as the basis of diagnosis and treatment. Canadian journal of gastroenterology = Journal canadien de gastroenterologie 15, 591–598 (2001). 11573102 10.1155/2001/367832

[b42] Xu Q. . Risk of gastric cancer is associated with the MUC1 568 A/G polymorphism. International journal of oncology 35, 1313–1320 (2009). 19885554 10.3892/ijo_00000449

[b43] Xu Q. . SNP-SNP interactions of three new pri-miRNAs with the target gene PGC and multidimensional analysis of H. pylori in the gastric cancer/atrophic gastritis risk in a Chinese population. Oncotarget 7, 23700–23714 (2016). 26988755 10.18632/oncotarget.8057 PMC5029657

[b44] Gong Y. H., Sun L. P., Jin S. G. & Yuan Y. Comparative study of serology and histology based detection of Helicobacter pylori infections: a large population-based study of 7,241 subjects from China. Eur J Clin Microbiol Infect Dis. 29, 907–911 (2010). 20440530 10.1007/s10096-010-0944-9

[b45] Liu R. . A five-microRNA signature identified from genome-wide serum microRNA expression profiling serves as a fingerprint for gastric cancer diagnosis. Eur J Cancer 47, 784–791 (2011). 21112772 10.1016/j.ejca.2010.10.025

[b46] Xu Q. . Expression of serum miR-20a-5p, let-7a, and miR-320a and their correlations with pepsinogen in atrophic gastritis and gastric cancer: a case-control study. BMC clinical pathology 13, 11 (2013). 23521833 10.1186/1472-6890-13-11 PMC3635921

[b47] Xu Q. . A New Polymorphism Biomarker rs629367 Associated with Increased Risk and Poor Survival of Gastric Cancer in Chinese by Up-Regulated miRNA-let-7a Expression. PLoS One 9, e95249 (2014). 24760009 10.1371/journal.pone.0095249 PMC3997364

[b48] Xu Q. . Promoter polymorphisms in trefoil factor 2 and trefoil factor 3 genes and susceptibility to gastric cancer and atrophic gastritis among Chinese population. Gene 529, 104–112 (2013). 23933418 10.1016/j.gene.2013.07.070

