# Supplementary material for: MiR-27a rs895819 is involved in increased atrophic gastritis risk, improved gastric cancer prognosis and negative interaction with Helicobacter pylori
Source: Sci Rep. 2017 Feb 2;7:41307. doi: 10.1038/srep41307 (PMC5288699; doi:10.1038/srep41307)
Supplement: Supplementary Dataset 1 [file srep41307-s1.doc]

***MiR-27a* rs895819 is involved in increased atrophic gastritis risk, improved gastric cancer prognosis and negative interaction with *Helicobacter pylori***

Qian Xu^1^, Tie-jun Chen^1^, Caiyun He^2^, Liping Sun^1^, Jing-wei Liu^1^, Yuan Yuan^1^,*

^1^Tumor Etiology and Screening Department of Cancer Institute and General Surgery, the First Affiliated Hospital of China Medical University, and Key Laboratory of Cancer Etiology and Prevention (China Medical University), Liaoning Provincial Education Department, Shenyang 110001, China

^2^Department of Molecular Diagnostics, Sun Yat-Sen University Cancer Center, State Key Laboratory of Oncology in South China, Collaborative Innovation Center for Cancer Medicine, Guangzhou, China

***Corresponding author:** Dr. Yuan Yuan, Tumor Etiology and Screening Department of Cancer Institute and General Surgery, North Nanjing Street 155#, Heping District, Shenyang110001, China

Telephone：+86-024-83282153; fax: +86-024-83282383. Email：[yyuan@cmu.edu.com.cn](mailto:yyuan@cmu.edu.com.cn)

**Supplementary Figure S1**: The selection of cell lines for pCMV-miR-27a-A or G plasmid transfection


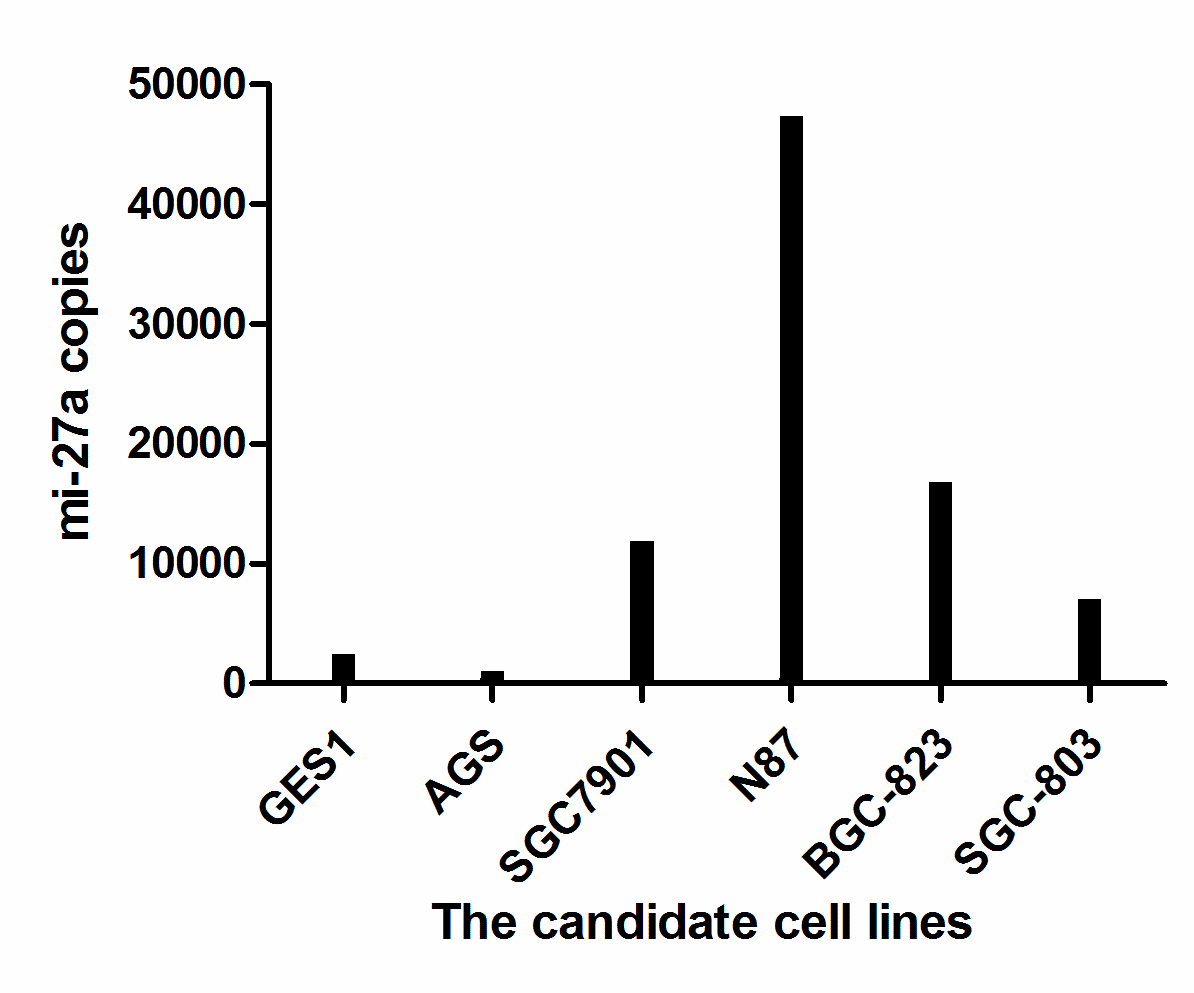


Figure Legend: The selection of cell lines for pCMV-miR-27a-A or G plasmid transfection. This figure showed endogenous miR-27a expressed by different cell lines (GES-1, AGS, SGC-7901, N87, BGC-823, SGC-803). The least cell line expressed endogenous miR-27a, AGS, was selected for transfection because it could reduce the effect that endogenous miR-27a participated in the transfection experiments on the other hand could warrant miR-27a upstream or downstream pathway molecules existed.

| Supplementary Table S1 The baseline of the subjects for the different gastric diseases | | | | | |
| --- | --- | --- | --- | --- | --- |
| Variables | AG vs. CON | |  | GC vs. CON | |
|  | CON(%) | AG(%) |  | CON(%) | GC(%) |
|  | **n=1166** | **n=1067** |  | **n=1033** | **n=939** |
| **Stage 1(Direct Sequencing)** |  |  |  |  |  |
|  | **n=304** | **n=205** |  | **n=304** | **n=215** |
| Gender | *P*=0.921 | |  | *P*=0.166 | |
| Male | 187(61.5) | 127(62.0) |  | 187(61.5) | 145(67.5) |
| Female | 117(38.5) | 78(38.0) |  | 117(38.5) | 70(32.6) |
| Age | *P*=0.061 | |  | *P*=0.074 | |
| Mean±SD | 55.37±11.11 | 57.32±12.14 |  | 55.37±11.11 | 57.16±11.44 |
| Median | 55 | 57 |  | 55 | 57 |
| Range | 23-81 | 28-83 |  | 23-81 | 21-80 |
| *H.pylori* | *P*=0.001 | |  | *P*=0.001 | |
| positive | 100(32.9) | 97(47.3) |  | 100(32.9) | 102(47.4) |
| negative | 204(67.1) | 108(52.7) |  | 204(67.1) | 113(52.6) |
|  |  |  |  |  |  |
| **Stage 2(Sequenom MassArray)** |  |  |  |  |  |
|  | **n=862** | **n=862** |  | **n=729** | **n=724** |
| Gender | *P*=0.846 | |  | *P*=0.564 | |
| Male | 483(56.0) | 487(56.5) |  | 483(66.3) | 490(67.7) |
| Female | 379(44.0) | 375(43.5) |  | 246(33.7) | 234(32.3) |
| Age | *P*=0.343 | |  | *P*=0.562 | |
| Mean±SD | 54.9±9.2 | 55.4±9.5 |  | 56.1±9.2 | 56.4±9.8 |
| Median | 54 | 56 |  | 56 | 57 |
| Range | 17-85 | 16-79 |  | 17-85 | 21-81 |
| *H.pylori* | *P*＜0.001 | |  | *P*＜0.001 | |
| Positive | 241(28.0) | 505(58.6) |  | 201(27.6) | 369(51.0) |
| Negative | 621(72.0) | 365(41.4) |  | 528(72.4) | 355(49.0) |
| Smoking | **n=586** | **n=548** |  | **n=500** | **n=333** |
|  | *P*=0.299 | |  | *P*=0.183 | |
| Ever Smoker | 202(34.5) | 173(31.6) |  | 199(39.8) | 148(44.4) |
| Never Smoker | 384(65.5) | 375(68.4) |  | 301(60.2) | 185(55.6) |
| Drinking | **n=585** | **n=547** |  | **n=499** | **n=296** |
|  | *P*=0.333 | |  | *P*=0.044 | |
| Drinker | 147(25.1) | 124(22.7) |  | 146(29.3) | 107(36.1) |
| Nondrinker | 438(74.9) | 423(77.3) |  | 353(70.7) | 189(63.9) |
| Note: CON, controls; AG, atrophic gastritis; GC, gastric cancer. | | | | | |

| Supplementary Table S2 The baseline of the subjects for the different area in Stage 2 | | | | | | | | |
| --- | --- | --- | --- | --- | --- | --- | --- | --- |
| Variables | CON(%) | |  | AG(%) | |  | GC(%) | |
|  | Zhuanghe | Shenyang |  | Zhuanghe | Shenyang |  | Zhuanghe | Shenyang |
|  |  |  |  |  |  |  |  |  |
| Gender | *P=*0.274 | |  | *P=*0.376 | |  | *P=*0.238 | |
| Male | 399(56.9) | 84(52.2) |  | 385(57.3) | 102(53.7) |  | 32(60.4) | 458(68.3) |
| Female | 302(43.1) | 77(47.8) |  | 287(42.7) | 88(46.3) |  | 21(39.6) | 213(31.7) |
|  |  |  |  |  |  |  |  |  |
| Age | *P=*0.513 | |  | *P=*0.001 | |  | *P=*0.237 | |
| Mean±SD | 56.3±9.9 | 54.5±10.0 |  | 54.6±9.0 | 58.1±10.7 |  | 58.0±9.7 | 56.3±9.9 |
| Median | 57 | 53 |  | 55 | 58 |  | 58 | 57 |
| Range | 21-81 | 34-84 |  | 28-79 | 16-77 |  | 34-80 | 21-81 |
|  |  |  |  |  |  |  |  |  |
| Rs895819 | *P=*0.155 | |  | *P=*1.000 | |  | *P=*0.884 | |
| AA | 397(56.6) | 85(52.8) |  | 364(54.2) | 103(54.2) |  | 26(49.1) | 343(51.1) |
| AG | 268(38.2) | 72(44.7) |  | 262(39.0) | 74(38.9) |  | 23(43.4) | 288(42.9) |
| GG | 36(5.1) | 4(2.5) |  | 46(6.8) | 13(6.8) |  | 4(7.5) | 40(6.0) |
| Note: CON, controls; AG, atrophic gastritis; GC, gastric cancer. | | | | | | | | |

| Supplementary Table S3 The baseline of the subjects for the intestinal-type GC and diffuse-type GC risk | | | | | |
| --- | --- | --- | --- | --- | --- |
| Variables | Intestinal-type GC vs CON | |  | Diffuse-type GC vs CON | |
|  | CON(%) | Intestinal-type GC(%) |  | CON(%) | Diffuse-type GC(%) |
|  | n=729 | n=316 |  | n=729 | n=457 |
| Gender | *P*=0.244 | |  | *P*=0.498 | |
| Male | 483(66.3) | 221(69.9) |  | 483(66.3) | 294(64.3) |
| Female | 246(33.7) | 95(30.1) |  | 246(33.7) | 163(35.7) |
| Age | *P<*0.001 | |  | *P*=0.105 | |
| Mean±SD | 56.14±9.21 | 59.09±9.37 |  | 56.14±9.21 | 55.19±10.78 |
| Median | 56 | 59.5 |  | 56 | 56 |
| Range | 17-85 | 21-80 |  | 17-85 | 26-81 |
| *H.pylori* | *P<*0.001 | |  | *P<*0.001 | |
| positive | 201(27.6) | 163(51.6) |  | 201(27.6) | 234(51.2) |
| negative | 528(72.4) | 153(48.4) |  | 528(72.4) | 223(48.8) |

| Supplementary Table S4 Association of miR-27a rs895819 polymorphism with the risk of intestinal and diffuse-type gastric cancer^a^ | | | | | | | | |
| --- | --- | --- | --- | --- | --- | --- | --- | --- |
| Variables | CON(%) | Intestinal-type GC(%) | Diffuse-type GC(%) | Intestinal-type GC vs CON | |  | Diffuse-type GC vs CON | |
|  |  |  |  | OR（95%CI） | *P* |  | OR（95%CI） | *P* |
|  | n=729 | n=316 | n=457 |  |  |  |  |  |
| AA | 411(56.4) | 154(48.7) | 237(51.9) | 1(Ref) |  |  | 1(Ref) |  |
| AG | 282(38.7) | 141(44.6) | 196(42.9) | 1.30(0.97-1.73) | 0.078 |  | 1.17(0.91-1.50) | 0.232 |
| GG | 36(4.9) | 21(6.6) | 24(5.3) | 1.53(0.84-1.78) | 0.168 |  | 1.16(0.66-2.03) | 0.600 |
| GG+AG VS. AA |  |  |  | 1.32(1.00-1.74) | 0.050 |  | 1.16(0.91-1.48) | 0.224 |
| GG VS. AG+AA |  |  |  | 1.33(0.74-2.37) | 0.343 |  | 1.07(0.62-1.84) | 0.823 |
| Note:^a^ using Logistic Regession adusted by gender, age and *H.pylori* infection status. CON:controls; GC:gastric cancer. | | | | | | | | |

| Supplementary Table S5 The heterogeneity test for the host's characteristics in the different stage | | |
| --- | --- | --- |
|  | Atrophic gastritis vs. control group | Gastric cancer vs. control group |
|  | *P*_heterogeneity_ | *P*_heterogeneity_ |
| Gender |  |  |
| Stage 2 vs. Stage 1 | 0.999 | 0.887 |
| Age |  |  |
| Stage 2 vs. Stage 1 | 0.095 | 0.812 |
| *H.pylori* |  |  |
| Stage 2 vs. Stage 1 | 0.985 | 0.078 |

| Supplementary Table S6 The association between the genotype of miR-27a rs895819 SNP and clinicopathological parameters^a^ | | | | | | | |
| --- | --- | --- | --- | --- | --- | --- | --- |
| Parameter | Genotype | | | AG vs. AA | GG vs. AA | Dominant model | Recessive model |
|  | AA | AG | GG | *P-value* | *P-value* | *P-value* | *P-value* |
| Age |  |  |  | 0.445 | 0.633 | 0.590 | 0.503 |
| ≤60 | 82(45.1) | 71(49.3) | 10(40.0) |  |  |  |  |
| ＞60 | 100(54.9) | 73(50.7) | 15(60.0) |  |  |  |  |
| Gender |  |  |  | 0.594 | 0.252 | 0.893 | 0.186 |
| Male | 131(71.2) | 110(73.8) | 15(60.0) |  |  |  |  |
| Female | 53(28.8) | 39(26.2) | 10(40.0) |  |  |  |  |
| Macroscopic type |  |  |  | 0.801 | 0.861 | 0.864 | 0.810 |
| Borrmann Ⅰ-Ⅱ | 37(22.4) | 31(23.7) | 5(20.8) |  |  |  |  |
| Borrmann Ⅲ-Ⅳ | 128(77.6) | 100(76.3) | 19(79.2) |  |  |  |  |
| Lauren grade |  |  |  | 0.700 | 0.553 | 0.605 | 0.604 |
| Intestinal | 69(37.5) | 53(35.6) | 8(32.0) |  |  |  |  |
| Diffuse | 112(60.9) | 94(63.1) | 17(68.0) |  |  |  |  |
| Unclassified | 3(1.6) | 2(1.3) | 0 |  |  |  |  |
| TNM stage |  |  |  | 0.873 | 0.375 | 0.909 | 0.342 |
| Ⅰ-Ⅱ | 91(49.5) | 75(50.3) | 10(40.0) |  |  |  |  |
| Ⅲ-Ⅳ | 93(50.5) | 74(49.7) | 15(60.0) |  |  |  |  |
| Depth of invasion |  |  |  | 0.748 | 0.731 | 0.854 | 0.663 |
| T1+T2 | 43(29.7) | 34(27.9) | 7(33.3) |  |  |  |  |
| T3+T4 | 102(70.3) | 88(72.1) | 14(66.7) |  |  |  |  |
| Lymphatic metastasis |  |  |  | 0.948 | 0.686 | 0.860 | 0.689 |
| Positive | 110(59.8) | 89(60.1) | 16(64.0) |  |  |  |  |
| Negative | 74(40.2) | 59(39.9) | 9(36.0) |  |  |  |  |
| Note: ^a^, using χ^2^ two-side test. | | | | | | | |

| Supplementary Table S7 The association between the clinicopathological parameters and the patients' survival | | | | |
| --- | --- | --- | --- | --- |
|  | All GC | Death | MST^a^ |  |
| Factors | n=357 | n=89 | (M) | *P* |
| Age |  |  |  | 0.614 |
| ≤60 | 168 | 41 | 59.2^b^ |  |
| ＞60 | 189 | 48 | 57.1^b^ |  |
| Gender |  |  |  | 0.797 |
| Male | 255 | 63 | 60.8^b^ |  |
| Female | 102 | 26 | 45.7^b^ |  |
| Smoking^c^ |  |  |  | 0.863 |
| Ever Smoker | 108 | 24 | 30.5^b^ |  |
| Never Smoker | 178 | 39 | 29.8^b^ |  |
| Drinking^c^ |  |  |  | 0.631 |
| Drinker | 90 | 18 | 29.8^b^ |  |
| Nondrinker | 196 | 45 | 29.9^b^ |  |
| Family history^c^ |  |  |  | 0.102 |
| Yes | 39 | 6 | 33.6^b^ |  |
| No | 247 | 57 | 29.5^b^ |  |
| *H. pylor*i-IgG^c^ |  |  |  | 0.992 |
| Positive | 131 | 31 | 56.2^b^ |  |
| Negative | 140 | 32 | 59.8^b^ |  |
| Macroscopic type |  |  |  | 0.055 |
| Borrmann Ⅰ-Ⅱ | 83 | 23 | 66.5^b^ |  |
| Borrmann Ⅲ-Ⅳ | 274 | 66 | 53.8^b^ |  |
| Lauren classification |  |  |  | 0.234 |
| Intestinal | 120 | 25 | 59.9^b^ |  |
| Diffuse | 231 | 61 | 59.3^b^ |  |
| Unclassified | 6 |  |  |  |
| TNM stage |  |  |  | **9.40×10^-15^** |
| Ⅰ-Ⅱ | 171 | 12 | 73.5^b^ |  |
| Ⅲ-Ⅳ | 186 | 77 | 27.0 |  |
| Depth of invasion |  |  |  | **5.35×10^-9^** |
| T1+T2 | 98 | 3 | 76.8^b^ |  |
| T3+T4 | 259 | 86 | 52.2^b^ |  |
| Lymphatic metastasis |  |  |  | **1.82×10^-8^** |
| Positive | 216 | 77 | 32.0 |  |
| Negative | 141 | 12 | 71.5^b^ |  |
| Note: a, MST, median survival time (months). ^b^, mean survival time was provided when MST could not be calculated. ^c^, parts of the data were missing, thus this factor could not be an adjusted factor for the analysis of miR-27a polymorphism and gastric cancer survival. | | | | |
